# The Progress of Mitophagy and Related Pathogenic Mechanisms of the Neurodegenerative Diseases and Tumor

**DOI:** 10.1155/2015/543758

**Published:** 2015-12-08

**Authors:** Ying Song, Wei Ding, Yan Xiao, Kong-jun Lu

**Affiliations:** Department of Pharmacology, Zhejiang University of Technology, Hangzhou Chaowang Road 18, Zhejiang 310014, China

## Abstract

Mitochondrion, an organelle with two layers of membrane, is extremely vital to eukaryotic cell. Its major functions are energy center and apoptosis censor inside cell. The intactness of mitochondrial membrane is important to maintain its structure and function. Mitophagy is one kind of autophagy. In recent years, studies of mitochondria have shown that mitophagy is regulated by various factors and is an important regulation mechanism for organisms to maintain their normal state. In addition, abnormal mitophagy is closely related to several neurodegenerative diseases and tumor. However, the related signal pathway and its regulation mechanism still remain unclear. As a result, summarizing the progress of mitophagy and its related pathogenic mechanism not only helps to reveal the complicated molecular mechanism, but also helps to find a new target to treat the related diseases.

## 1. Introduction

Mitochondria are a kind of semiautonomous organelle which contain two layers of membrane, which are the main places for cells to operate the oxidative reaction and produce the adenosine triphosphate (ATP). Mitochondria are called “the energy center of the cell” and can be divided into outer membrane, inner membrane, and membrane space. They are also involved in the cellular differentiation, proliferation, and apoptosis.

Although the aerobic oxidation is much more efficient than anaerobic glycolysis, oxidative phosphorylation will also produce reactive oxygen species (ROS). High levels of ROS will damage mitochondria and release the proapoptosis factors that finally induce the death of cell [1]. The oxidative phosphorylation mainly takes place on the electron transport chain of the mitochondrial membrane. However, when a large number of electronics are transferred by the chain, it will cause the reduction of oxygen to ROS. As a result, the energy produce efficiency will decrease with the production of the ROS. It is well known that mitochondrial damage is the major consequence of oxidative stress because of the high dependence of mitochondrial function on redox-sensitive targets such as intact membranes. Even under normal circumstances, some of the mitochondria would be damaged because of the accumulation of ROS. Therefore, removing the damaged mitochondria is necessary for cells to maintain their normal state. Lemasters's study firstly showed that the renewal of mitochondria is mainly through the selective autophagy, whose process of removing mitochondria has been known as mitophagy. Chen's team has reported a new regulatory molecular mechanism of mitophagy in mammalian cells; they found the switch of mitochondria could promote the cancer metastasis [2]. It has reached a consensus that abnormal mitophagy contributes to several neurodegenerative diseases, diabetes, and tumor. Here, this review summarizes the studies of mitophagy and its related pathogenic mechanisms.

## 2. Mitochondria and Mitophagy

In eukaryotic cell's regulatory network, mitochondrion is one of the most important organelles. In addition to supplying cellular energy, it is also involved in cellular differentiation, proliferation and apoptosis. When a variety of stimulations such as ultraviolet rays, oxidative stress, and virus are gathered in mitochondria, mitochondria will get destroyed. Then, the mitochondrial permeability will change or even collapse. Finally, cell starts apoptosis.

**Figure 1:**
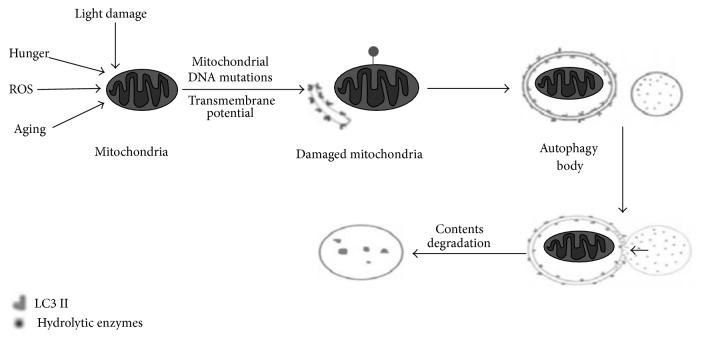
The diagram of mitophagy process [5]. Under stimuli like ROS, lack of nutrition, and cell aging, mitochondria underwent depolarization and then damaged themselves; the damaged mitochondria were packaged into autophagy and fused with lysosomes and then completed the degradation process and maintained the stability of the internal environment.

Autophagy is the main approach for metabolizing mitochondria [3]; the process of mitochondrial removal through autophagy is called mitophagy. As shown in Figure 1, stimulated by factors such as ROS and lack of nutrition, mitochondrion will become depolarized and then damage itself. The damaged mitochondria are packaged into autophagosome and fused with lysosomes, so as to complete the degradation process and maintain the stability of the internal environment [4].

The original process of mitophagy requires two steps: the general induction and the specific recognition. As for general induction state, the related proteins are also involved in inhibiting the mammalian target of rapamycin (mTOR) induced by ROS (which is produced by the damaged mitochondria) and activating AMPK caused by the loss of ATP. The signal transduction pathway includes dependent PI3K/Akt-I and nondependent PI3K/Akt-I pathway. The dependent PI3K/Akt-I pathway is a classical pathway of mTOR involved in autophagy. When Protein Tyrosine Kinase (PTK) and G Protein-Coupled Receptors (GPCRs) located on the cell surface and combined with the ligands outside the cell, which can isolate the regulatory subunit of PI3K and release its catalytic subunit, then translate phosphatidylinositol 4,5-bisphosphate (PIP2) into phosphatidylinositol 3,4,5-triphosphate (PI3P) by phosphorylation. PI3P is an important signaling molecule. It is able to combine with many proteins, which have homologous substrate, such as phosphoinositide-dependent kinase 1 (PDK1) and Akt. PDK1 combines with PI3P on the cell membrane and phosphorylates Akt. The activated Akt phosphorylated many target proteins in damaged mitochondria and inhibited the mTORC1 from activating and then promoted the occurrence of autophagy and cleared the mitochondria. The nondependent PI3K/Akt signaling pathway is activated by the loss of mitochondrial ATP, which can activate the liver kinase B1 (LKB1) or AMP activated protein kinase (AMPK) signaling pathway and affect the activity of mTORC1. After the activation of AMPK, the activated AMPK can phosphorylate tuberous sclerosis complex 2 (TSC2) directly, enhance the activity of GTPase-activating protein (GAP), and translate the ras homolog enriched in brain-GTP (Rheb-GTP) into the ras homolog enriched in brain-GDP (Rheb-GDP), which clears the mitochondria by inhibiting mTORC1 and activating autophagy. As for the specific recognition, it is involved in multiple mechanisms, including the Parkin dependence process mediated by PINK1-Parkin signaling pathway and the Parkin independence process mediated by Nix and Bnip3 [6].

## 3. Mitophagy and Abnormal Mitophagy

### 3.1. Mitophagy Types

**Figure 2:**
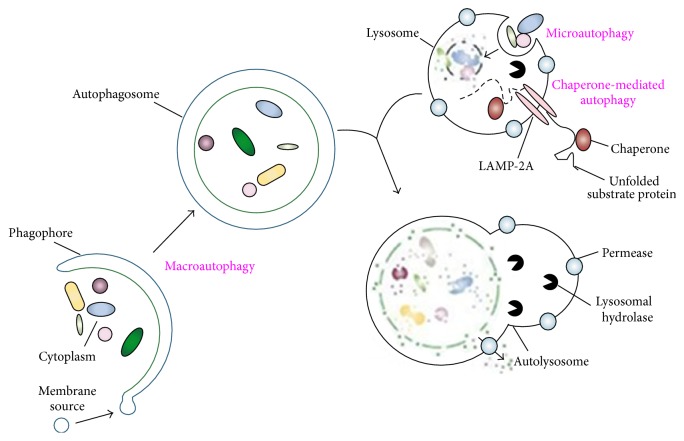
The type of autophagy and its basic process [8]. (1) Microautophagy refers to the sequestration of cytosolic components directly by lysosomes through invaginations in their limiting membrane. Microautophagy-like processes in fungi are involved in selective organelle degradation. (2) In the case of macroautophagy, the cargoes are sequestered within a unique double-membrane cytosolic vesicle, an autophagosome. The autophagosome is formed by expansion of the phagophore. (3) Lysis of the autophagosome inner membrane and breakdown of the contents occur in the autolysosome, and the resulting macromolecules are released back into the cytosol through membrane permeases. CMA involves direct translocation of unfolded substrate proteins across the lysosome membrane through the action of a cytosolic and lysosomal chaperone hsc70 and the integral membrane receptor LAMP-2A.

Depending on the different ways of substrates' transporting to lysosomes, autophagy can be classified into three types: macroautophagy, microautophagy, and cytosol-to-vacuole transport (Cvt). Some people think Cvt should be replaced by chaperone-mediated autophagy [7]. The type of autophagy and its basic process are shown in Figure 2.

In macroautophagy, autophagy body wraps the mitochondria by forming a double-membrane and then fuses with lysosomes to generate autophagy-lysosome which finally degrades the mitochondria. Microautophagy means directly completing the mitochondrial degradation by the phagocytosis of lysosomes. Autophagy mediated by molecular chaperone is a process in which the target protein combines with the partner protein and is transported through the lysosomal membrane. LAMP-2A (which is the lysosome's related membrane protein) recognizes these partner proteins and starts the degradation process. Cvt process only exists in yeast; it has not yet been found in mammalian cells [1].

### 3.2. The Components of the Mitophagy and Their Role

Evidence has shown that mitophagy may not simply be induced by the mitochondrial damage. It is also related to nonspecific regulation of autophagy. The defection of mitophagy genes such as Atg32 and Atg11 will reduce or even block the activity of autophagy, which means that the general response of autophagy induced by mitochondrial damage will be regulated by mitophagy proteins [9]. In the molecular mechanisms, almost the whole set of Atg proteins are involved in microautophagy. Atg32, located on the mitochondrial outer membrane, participates in Cvt and mediates the mitochondria into autophagosome to decompose through two ways: (1) acting as mitochondrial receptor and combining with Atg11 and (2) interacting with Atg8 because it has the same binding motifs with Atg8. Uth1 and Aup1 are mitochondrial proteins which mediate mitophagy. Located on the mitochondrial outer membrane, Uth1 regulates the pathway of microautophagy specifically and is expressed constitutively under different growth condition. Mutation of Uth1 will lead to the dysfunction of mitochondrial clearance [10]. Aup1 is one of the phosphatase analogues in the yeast and interacts with Atg1p which is an autophagy related protein kinase. In the cells with mutational Aup1, the mitophagy level and cell's viability decrease significantly. NIP3(NIX) located on the mitochondrial outer membrane, which mediates the pathway of macroautophagy and participates in the removal process of mitochondria during the maturation of red blood cells. As the mitochondrial receptor, NIX combines with the GABA receptor (which related to LC3 and its homologue) through WXXL motif and mediates mitochondria into autophagosome [11].

PINK1, which helps to maintain the normal function of organelles through removing the damaged mitochondria selectively, is a protein kinase on the mitochondrial outer membrane. PINK1 is transported to all mitochondria after its expression. PINK1 will be degraded by proteolytic enzymes when it reaches normal mitochondria, while it will continue to accumulate in damaged mitochondria. The inhibition of proteolytic enzymes activity will induce the occurrence of mitophagy and the cell dies in the end. When mitochondrial fusion is blocked, it will cause the damage of mitochondria and the loss of membrane potential. E3 ubiquitin ligase (Parkin) will be transported to the damaged mitochondria selectively. The normal mitochondria do not have Parkin's position; it is only expressed in damaged mitochondria. The process in which Parkin is transported to the damaged mitochondria and induces mitophagy requires the help of PINK1. PINK1 and Parkin are vital to mediate the degradation process of damaged mitochondria. Recent studies have shown that PINK1 and Parkin mediated the mitophagy through three stages: transportation of Parkin to the damaged mitochondria, formation of mito-aggresomes, and the degradation of mitochondria [12]. On the one hand, PINK1 will be degraded on the normal mitochondria rapidly, and the damaged mitochondria make the PINK1 accumulate effectively and then induce mitophagy. On the other hand, PINK1 recruits Parkin to the damaged mitochondria, strengthening the activity of its E3 ubiquitin ligase, which makes the mitochondria matrix protein ubiquitin. Then, P62 accumulates on the mitochondria matrix, combines with LC3, and mediates the ubiquitin's substrate into autophagosome, so the joint action of PINK1 and Parkin causes the damaged mitochondria to be selectively cleared by autophagy in the form of complete organelles [13].

**Figure 3:**
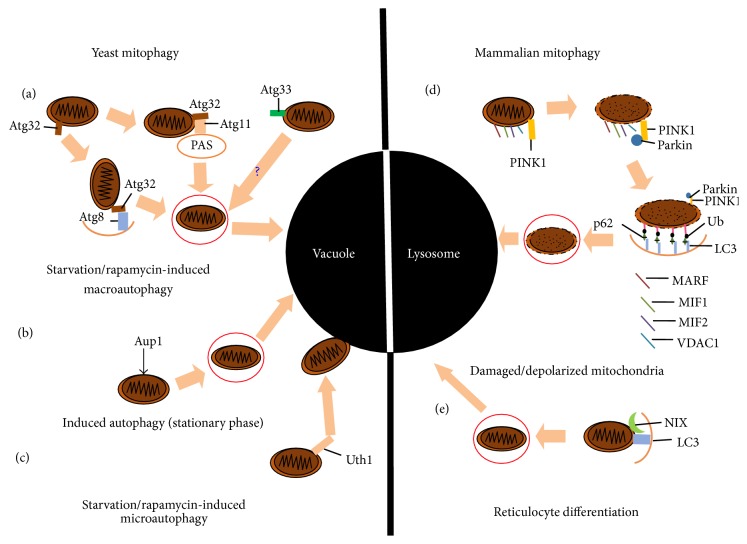
The components and machinery of the mitophagy [14]. (1) In yeast, three examples of mitophagy are represented: (a) the classic starvation/rapamycin-induced pathway is mediated by TOR and results in macroautophagy. Atg8 interacts with Atg32, which in turn interacts with Atg11 at the PAS and engulfs and delivers the mitochondria to the vacuole. (b) Induced autophagy in the stationary phase. This pathway relies on the autophagosomal identification of mitochondria via the Aup1 and results in macroautophagic disposal; (c) starvation and rapamycin can also induce macroautophagy, a process in which recognition of Uth1 results in the direct engulfment of mitochondria by the vacuole. (2) Two further examples of mitophagy in mammals are represented: (d) MARF, MIF1, and PINK1 localizes on the mitochondrial outer membrane and interacts with Parkin and leads to the polyubiquitination of damaged and/or depolarized mitochondria, which ultimately leads to the macroautophagic engulfment and delivery of the mitochondria to the lysosome for destruction; (e) reticulocytes remove mitochondria through NIX recognition by the mammalian Atg8 homologue, LC3.

The discovery of autophagy and a large number of related experiments were initially carried out on the yeast, which identified many autophagy genes (Atg). Later, the homologues of Atg were found in mammals. Under normal circumstances, cytokines activated the PI3K-I proteins which in turn activated mTOR through Akt signal pathway. The activated mTOR inhibits the occurrence of autophagy by inhibiting Atg1 which is the key signaling molecule of autophagy. Under stress circumstances, mTOR is not activated, and Atg1 forms the complex which acts as a signal for the induction of autophagy by raising Atg11, Atg13, and Atg17. Moreover, activating the signal requires another two complexes: one is composed of Atg6 (Beclin1), PI3K-III, and Atg14; another which is the key to raise Atg8 (LC3) is composed of Atg12, Atg16, Atg15, and Atg7. In the induction of autophagy, the LC3-I in the cytoplasm is cleaved and becomes LC3-II. The homologues of Atg1 in mammals include unc-51-like kinase1/2 (ULK1/2), three proteins (KIAA0652, FIP200, and Atg101) that interact with ULK1/2. The complex of PI3K-I (including Vps34, Vps15, Vps30/Atg6, and Atg14) is necessary for autophagy. The homologues of PI3K-I in mammals include Vps34, p150, Beclin1, Atg14, and ultraviolet irradiation resistance genes. Nix is a kind of binding protein which is necessary for mitophagy in the development of red blood cells. Nix in mammals may have a similar function as Atg32 in yeast because they have the same domains. The components and machinery of the mitophagy are shown in Figure 3.

### 3.3. Different Levels of Mitophagy Produce Different Results

Because the unpolarizing mitochondria can activate the apoptosis pathway by releasing apoptosis proteins if cells remove the damaged mitochondria by autophagy, mitophagy may act as a protective mechanism. However, excessive autophagy may induce the cathepsin or other proteolytic enzymes to leak from lysosomes or autophagy-lysosomes and then cause type II programmed cell death.

When the outside stimuli are weak, mitophagy acts as a protective mechanism. When the outside stimuli are strong and mitophagy is not powerful enough to remove the damaged mitochondria, it will induce apoptosis. When the strong stimuli make the mitochondrial membrane's permeability change greatly, the amount of ATP sharply declines and the cellular energy system is destroyed completely. As a result, cells cannot provide the energy for autophagy or apoptosis. The strong stimuli finally lead to cell necrosis [5]. Both the deficiency and the immoderacy of mitophagy are the manifestation of abnormal mitophagy.

Although autophagy is another kind of programmed cell death, it is different from apoptosis. Under specific conditions, mitophagy acts as an inducer that triggers cell death. Apoptosis and necrosis are two different kinds of cell death procedures. They can be transformed into each other in certain conditions and may cause the activation of a series of protein hydrolyses in the same family. Cells have a universal pattern in the process of cell death under pathological conditions. The nuclear debris produced in the process of cell death also exists in necrosis and cell death induced by mitophagy. Therefore, the occurrence of mitophagy is closely related to apoptosis and necrosis, which have different effects in different stages of the disease.

After mitophagy, the abnormal mitochondria are cleared, the stability of cell is maintained, and necrosis is prevented. Therefore, mitophagy is very important for preventing some diseases, such as neurodegenerative diseases, cancer, and immune system diseases. The deficiency of mitophagy disables cells to clear the damaged mitochondria in time, so the damaged mitochondria accumulate in cell and undergo apoptosis. The immoderacy of mitophagy may eliminate too many mitochondria, so that cells cannot maintain basic functions and undergo cell necrosis. Both the deficiency and the immoderacy of mitophagy can lead to the occurrence of disease.

## 4. Mitophagy and Neurodegenerative Diseases

Mitophagy is vital for maintaining the normal function of neurons and usually acts as a protective role for cells, which also helps cells to remove the damaged mitochondria. Schaeffer's study has found that drugs can significantly relieve the symptoms of neurodegenerative disease by activating autophagy [15]. Atg6 (Beclin1) is an autophagy protein, whose expression in brain will decrease with the increase of age. It indicates that, with the increase of age, the level of autophagy slowly decreases, which finally leads to the increased incidence of neurodegenerative diseases [16]. Recently, it has been found that mitophagy is involved in several neurodegenerative diseases, including Parkinson's disease, Alzheimer's disease, and Huntington's disease.

The accumulation of abnormal proteins in neurons and other types of nerve cells usually results in neurodegenerative diseases because of their toxicity, which may interfere with nerve cell's normal function, for example, *α*-synuclein in Parkinson's disease, Tau in Alzheimer's disease, and Huntingtin in Huntington's disease [17]. Autophagy's physiological function is to degrade these abnormal proteins' aggregation. In recent years, more and more researches suggested that the basic level of autophagy is significant to remove the abnormal proteins' aggregation and maintain the nerve cells' normal function. During that process, mitophagy proteins also have effects that cannot be neglected. For example, using the mouse nerve cells which are deficient in Atg5 or Atg7 for research, researchers have found that the nerve cells lost the progressive movement function and the inclusion body appeared in the cytoplasm of neuron [18].

### 4.1. Mitophagy and Parkinson's Disease

Parkinson's disease (PD) is one of the typical neurodegenerative diseases caused by mitochondrial dysfunction [19]. People who have suffered PD usually have a large defection in nigra and amygdala. In PD patients, there are edematous mitochondria in nerve cells. It has been shown that the morbidity of PD maybe associated with the failure of damage mitochondrial removal [20]. Failing to remove the damaged mitochondria through mitophagy will lead to the impairment of mitochondria, cause oxidative stress reaction, and increase toxicity materials. It finally leads to dopaminergic neuron's death and causes PD [21].

Studies on the PD familial transmissibility have confirmed that mitophagy plays a dominant part in inherited and sporadic PD [22]. *α*-Synuclein is the key protein of PD and the main component of its lewy body. The abnormal *α*-synuclein results from duplicate expression and mutational gene, both of which can lead to PD [23]. The mutational *α*-synuclein will cause the formation of amyloid fibers which aggravate the disease directly. *α*-Synuclein inhibits autophagy through blocking LAMP-2A receptor (mediated by molecular chaperone) and stopping the transportation and degradation of proteins in lysosomes. The mutational cells compensate CMA's defection by raising the level of autophagy. But it is different from the normal protective role of autophagy. The raised compensatory autophagy induced by mutational nucleoprotein, especially mitophagy, which is harmful to cells, will lead to the escalation of familial and sporadic PD [24]. In addition, mitochondrial dysfunction transfers to autophagosome regulated by specific proteins (PINK1 and Parkin), so autophagy proteins are closely related to the occurrence of PD. Mutational PINK1, the autosomal recessive gene of PD, is involved in controlling autophagy. The PINK1 interacting with Beclin1 is stimulative to autophagy's occurrence while the mutational PINK1 interacting with Beclin1 plays an inhibitive role in autophagy's occurrence. Studies have shown that PINK1 could make the activity of Beclin1 decline only when its mutational site lied in W437X. Other mutational sites almost have no effect on the activity of Beclin1 [25]. Another autosomal recessive gene of PD is Parkin, which serves as a selective autophagy signal of dysfunctional mitochondria. Parkin also involves degrading the damaged mitochondria and acts as ubiquitin ligase E3. When it interacted with PINK1, Parkin can participate in mitophagy and clear the dysfunctional mitochondria. Researchers have found that, in the nuclei of substantia nigra brain nerves, as the age increased, the mitochondrial removal ability got a peak, which may lead to the occurrence of PD [26]. Even though the mouse (knocked out Parkin and PINK1) shows the defection of mitochondria and the activity of complex I decreases significantly, it does not develop PD ultimately. Studies on the cell culture system have confirmed that the activity of complex I decreases and the function of mitochondria is damaged in patients with mutational Parkin [27]. It is also determined that at least mitophagy's defection is one of the pathogeneses of PD. In a word, mitophagy has a close relationship with the occurrence of PD.

### 4.2. Mitophagy and Alzheimer's Disease

The progressive dementia and the transformed structure of cerebral morphology are the typical characteristics of Alzheimer's disease (AD), such as senile plaques and neurofibrillary tangles (NFTs). A*β* is the main component of senile plaques and phosphorylated Tau is the main component of the tangles. The main pathologies of AD are *β* amyloid deposited inside and outside the cell and tangles caused by the excessive phosphorylation of Tau. A series of continuous processes of APP's catabolism and A*β*'s formation are closely associated with AD. Since autophagy is involved in both A*β*'s production and elimination, there are disputes whether autophagy has double functions in the pathogenesis of AD [28]. In the brain of Alzheimer's patients, a large number of *β* amyloids accumulated in autophagy bubbles. Examinations of AD patient's brain show the AP's gather and mitochondrial dysfunction [16]. Cerebral biopsy specimens of Alzheimer's patients also show that cytolysosomes and other former lysosome vesicles increase significantly. All of them indicate that autophagy is crucial in the progression of disease. Mitochondrial DNA is much easier to mutate than nuclear DNA, so mitochondria are much easier to be damaged [29]. When mitochondrial DNA contains the engulfed mitochondria, the level of mtDNA is elevated in hippocampal neurons of AD. It prompts that the mitophagy's proportion increases in AD. Continuous analyses of ultrastructure of the AD neurons have found that most of the organelles are autophagosomes, which further supports the above conclusion.

Majority of researches have confirmed that the mature autophagy's defection is the common pathology characteristic of AD. Lysosome protease inhibitors and vincristine can prevent the AP's maturity and accumulate the immature autophagy bubbles. This explains that the defection of autophagy-lysosome can lead to the accumulation of large number of immature autophagy bubbles and the AP in brain. The mechanism may associate with the flow block of autophagy [30]. Because there almost have been no opportunities to see autophagosomes in healthy neurons, the damaged autophagy's AD flow may relate to the increased population of autophagosomes in AD neurons [31].

The decreased expression of Beclin1 in brain cells of AD and activated mitophagy proteins are the main contributions to AD. Studies have found that, in the transgenic mouse model of AD, enhanced Beclin1's expression will reduce the formation of *β* amyloid [32]. This also indicates that the expression level of Beclin1 is closely associated with the formation of *β* amyloid. Using medicine to inhibit Tau's phosphorylation or knocking out Tau can partly reverse the neurodegenerative symptoms of the missing Atg7 in mouse. This suggests that Atg7's defection has an important relationship with the accumulation of phosphorylated Tau in neurodegenerative disease [15]. Many studies have confirmed the above conclusion. In a word, mitophagy is associated with the occurrence of AD.

### 4.3. Mitophagy and Huntington's Disease

Huntington's disease (HD) is caused by mutational Huntingtin gene that occurred in the short arm of chromosome 4 p16.3. The main pathological changes are inclusion bodies and aggregations formed by mutational Htt (the main ingredients are fibrous proteins formed by Htt fragments which contain the prolonging polyQ) appeared in the central nervous system neurons, which leads to losing neurons, damages progressive cognition, and changes the sample involuntary movement. Studies of patients and mouse model of HD have found that the level of autophagy is positively correlated with the expression of Huntingtin. The aggregation and formation of inclusion body induced by mutational Htt made the neurons of cortex striatum degeneration and necrosis and eventually led to the mitochondrial dysfunction.

The identification deficiency of mitophagy substrates leads to the partial deficiency of Huntington [16]. Researches about the mouse, the cell lines, and patients of HD found that AP's formation and clearance were normal, but autophagy's inclusions were very rare, which indicated that the inclusion's defective identification resulted in the fact that the component could not be swallowed effectively in the cytoplasm, the abnormal mitochondria, and protein aggregated in the end. However, blocking mitophagy individually by lactacystin or knockout Beclin1 would cause the accumulation of Htt. Therefore, mitophagy is closely related to HD.

In summary, mitophagy is closely related to the occurrence and development of many kinds of neurodegenerative diseases. Mitophagy becomes one of the targets for the treatment of these diseases. Rapamycin is the first chemical medicine produced by researchers to treat the disease, which inhibits the activity of mTOR kinases that can inhibit mitophagy when they are activated [33]. Thus, rapamycin can activate mitophagy. However, the treatment by rapamycin is long-term and has side effects [34]. Therefore, researchers turned to research new drugs, such as lithium carbonate that increases the level of mitophagy. Recent studies have found that trehalose, which has protective effect on many kinds of cells, is a new type of mitophagy inducer [35]. It is very promising to develop trehalose into a clinical therapeutic agent. Nevertheless, it is still difficult to treat some neurodegenerative diseases by increasing the level of mitophagy. Therefore, studying mitophagy and its regulation mechanisms deeply is the key to find clinical therapeutic agents to treat the diseases.

## 5. Mitophagy and Tumor

**Figure 4:**
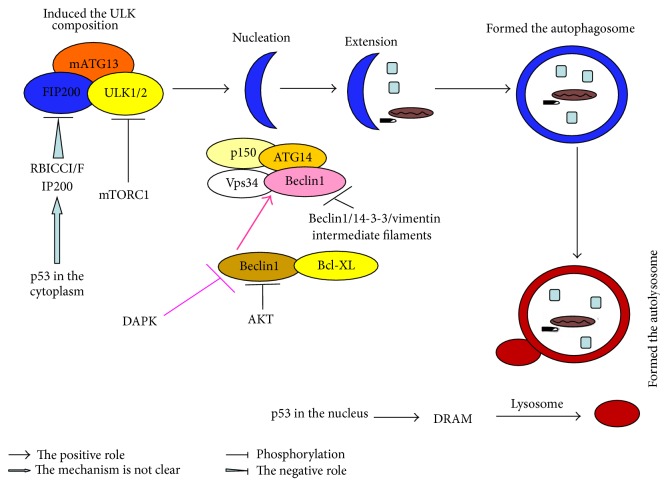
The formation of autophagy and its regulation mechanisms [36]. Autophagy is a multistep process involving induction, nucleation, extension, autophagosome's formation, and autophagy-lysosome's formation controlled by a set of ATGs. (1) The process starts with the activation of the ULK serine/threonine kinase complex that includes ATG13 and FIP200. This complex is regulated by mTOR which inhibits autophagy by phosphorylation of ULK1/2. The induction of autophagy is completed with the accumulation of the ULK1/2-ATG13-FIP200 complex, resulting in the phagophore. (2) Autophagosome's development is dependent on PI3K complex involving the proteins Vps-34, beclin1, and p150. This complex localizes to the phagophore and recruits further ATGs to allow for extension and completion of the autophagosome. ATG14 is the positive regulators binding to beclin1. The Rubicon molecule binds to beclin1 and reduces Vps34 activity and impairs autophagosomes formation as a negative regulator. Bcl-2 binds to the beclin1 BH3 domain also as a negative regulator. (3) Once the autophagosome is created, maturation is completed by fusion with a lysosome to form an autophagolysosome. This process involves LAMP1 and LAMP2, as well as UVRAG and the GTPase Rab7.

In the tumor's occurrence and development, autophagy is a multisteps dynamic process, including induction, nucleation, extension, formation of autophagosome, and autophagy-lysosome [36]. Besides autophagy proteins, the process is also affected by the regulation of a variety of signal molecules, such as mTOR, Beclin1, and p53 [37]. The formation of autophagy and its regulation mechanisms are shown in Figure 4.

Studies have shown that mitophagy is involved in the occurrence and development of tumor. There is single allelic loss of Beclin1 in breast cancer, ovarian cancer, and prostate cancer. Studies on the Beclin1-lacking mouse have found that the mouse was more likely to develop the non-Hodgkin's lymphoma, lung cancer, and breast cancer lesions. It has been confirmed that autophagy could inhibit a wide variety of tumors from the opposite side. Ubiquitin protein's aggregation is induced by the deficient autophagy, the aggregation of mitochondria and peroxisomes is induced by malfunction, and the escalation of oxidative stress's level and the activation of response are induced by DNA damage leading to the instability of gene, then increasing the occurrence of tumor. Studies have shown that the process was related to the sustained expression of p62 and the expression of NF-*κ*B regulated by p62 [38]. It was evidently shown that autophagy was a factor to inhibit the tumor. When ROS (produced by damaged organelles in the cell) impaired DNA and induced tumor, autophagy would be involved in clearing up the damaged mitochondria and peroxisomes, maintaining the cell function and the stability of chromosome, reducing the ROS's impairment, thus inhibiting the occurrence of tumor [39]. Most researchers thought that autophagy could promote the progression of tumor. Nutritional deficiencies and lack of oxygen are the common microenvironment of tumor. Autophagy induced by lacking of oxygen is beneficial to cell survival and promotes the progression of tumor. Fleming thought that the rapid growth of tumor cells would cause ischemia and hypoxia in the local tumor tissues. Tumor cells can respond to varieties of metabolic stress by inducing autophagy and inhibiting the growth of tumor cells at the same time. It was suggested that autophagy was productive in the growth of tumor cells [40] and the tumor microenvironment might also activate autophagy by independent HF-1 *α* mechanisms. Energy consumption of endoplasmic reticulum and catabolism omentum is greatly reduced while other organelles such as mitochondria gained the metabolic energy at the same time. It is beneficial to tumor cell's survival [41]. Other researches suggested that autophagy could promote the progress of tumor by the growth of mutational auxiliary gene. At present, more and more scholars tend to maintain the neutral attitude. Gong's studies have shown that high expression of autophagy proteins (Beclin1) could not only inhibit the growth of tumor cells, but also increase the aggregation of tumor stem cells [42]. Therefore, autophagy has double effects (promoting and inhibiting) on tumor's occurrence and development and it can be converted in some cases [43]. Besides, researches have shown that autophagy also played a dual role in the treatment of tumor. The mechanism might be linked to cell growth, death, and metabolism mediated by autophagy [44].

In 2011, Kitamura and Miyamoto researched the mitophagy system and they pointed out that mitophagy acted as a regulating mechanism in tumorigenesis [45, 46]. They thought that the vacuole of autophagy structure (MIV) mediated by Mieap might be associated with the classical macroautophagy. Nix and Mieap were important in this new discovery pathway called MALM. Generally, MALM did not occur before MIV; only when MALM was blocked or could not repair mitochondrial damage, it would start the MIV way to degrade the damaged mitochondria. Moreover, both of these systems accepted the regulation of p53 and ROS. In July 2014, the researchers of the Catholic University used mouse to study human tumors and they found the switch of mitochondria could promote the cancer metastasis. Mitochondria are the cellular energy center. Dysfunction of mitochondria in tumor cells could promote the migration of cell and eventually made the tumor cell spread successfully. Researchers have studied the molecular mechanisms involved in the process. It has been shown that, under certain conditions, mitochondria could produce more superoxide ions than usual. The excessive superoxide ions will trigger the formation of tumor's metastases and eventually form the tumor in the new organization [47].

Conventional treatments of tumor mainly include surgery, radiotherapy, and chemotherapy, but the treatment resistance usually occurs in the treatment, and surgery cannot improve the survival rate of patients very well. Consistent with its roles in the development of tumor, mitophagy also has the duplicity characteristic in the treatment of tumor. A variety of methods have been found to improve the therapeutic effect of tumor by regulating mitophagy. Increasing the level of mitophagy is one of these methods. If we combine mitophagy inducers with radiotherapy and chemotherapy, it will improve the effect of radiotherapy and chemotherapy [48]. On the other hand, decreasing the level of mitophagy is also one of these methods; mitophagy inhibitors also can improve the effect of radiotherapy and chemotherapy [49]. In addition, mitophagy can exert the role of anticancer through mediating immune responses [50]. With the development of science and the growing understanding of mitophagy, mitophagy will be well used to treat tumor.

## 6. Summary and Outlook

In conclusion, mitophagy is of great importance in scavenging the damaged mitochondria and maintaining cellular stable state. Mitophagy is closely linked to the occurrence and development of a variety of human diseases. Currently, mitophagy is one of the fastest development fields of cell biology's research direction. With in-depth studies in this field, new mitophagy pathways will continue to be found, which can put forward new problems and challenges for the further studies. Further study on the molecular mechanisms of mitophagy will substantially enhance the understanding of mitophagy related diseases such as neurodegenerative diseases, heart diseases, diabetes, and tumors. It will help provide new ideas, find new targets for the treatment of such diseases, and eventually help find efficient and suitable new drugs.
